# Safety and Efficacy of Robotic Radiosurgery for Visceral and Lymph Node Metastases of Renal Cell Carcinoma: A Retrospective, Single Center Analysis

**DOI:** 10.3390/cancers13040680

**Published:** 2021-02-08

**Authors:** Severin Rodler, Melanie Schott, Alexander Tamalunas, Julian Marcon, Annabel Graser, Jan-Niclas Mumm, Jozefina Casuscelli, Christian G. Stief, Christoph Fürweger, Alexander Muacevic, Michael Staehler

**Affiliations:** 1 Department of Urology, University Hospital of Munich, 80333 Munich, Germany; Melanie.schott@med.uni-muenchen.de (M.S.); Alexander.tamalunas@med.uni-muenchen.de (A.T.); julian.marcon@med.uni-muenchen.de (J.M.); annabel.graser@med.uni-muenchen.de (A.G.); Janniclas.Mumm@med.uni-muenchen.de (J.-N.M.); jozefina.casuscelli@med.uni-muenchen.de (J.C.); christian.stief@med.uni-muenchen.de (C.G.S.); michael.staehler@med.uni-muenchen.de (M.S.); 2 European CyberKnife® Center, 81377 Munich, Germany; Christoph.fuerweger@cyber-knife.net (C.F.); alexander.muacevic@cyber-knife.net (A.M.)

**Keywords:** robotic radiosurgery, renal cell carcinoma, metastatic disease

## Abstract

**Simple Summary:**

In metastatic renal cell carcinoma (mRCC), systemic treatment with checkpoint inhibitors or tyrosine kinase inhibitors is recommended in guidelines. However, the treatment of patients with oligometastatic disease or mixed responses remains challenging. We aimed to investigate the safety and efficacy of robotic radiosurgery in patients with mRCC. Sixty patients with visceral and lymph node metastases were selected for robotic radiosurgery. The median progression free survival of all patients was 17.4 months, local tumor control was achieved in 96.7% of patients, and only 8.3% of patients experienced adverse events. Robotic radiosurgery might be a powerful tool in addition to systemic treatment for patients with mRCC, but additive effects of both treatments require further investigation.

**Abstract:**

Despite rapid advances of systemic therapy options in renal cell carcinoma (RCC), local tumor or metastases treatment remains important in selected patients. Here, we assess the safety and efficacy of robotic radiosurgery (RRS) as an ablative therapy for visceral and lymph node metastases of RCC. Patients with histologically confirmed RCC and radiologically confirmed progression of visceral or lymph node metastases underwent RRS and were retrospectively analyzed. Overall survival and progression free survival were calculated by the Kaplan–Meier method and log-rank test. Sixty patients underwent RRS and were included in the analysis. Patients presented for RRS treatment with a median age at RRS treatment of 64 years (range 42–83), clear cell histology (88.3%) and favorable international metastatic renal cell carcinoma database (IMDC) risk score (58.3%). Treatment parameters differed for the number of fractions (median visceral metastases: 1, range 1–5; median lymph node metastases: 1, range 0–5; *p* = 0.003) and prescription dose (median visceral metastases 24 Gy, range 8–26; median lymph node metastases 18 Gy, range 7–26, *p* < 0.001). The median overall survival was 65.7 months (range: 2.9–108.6), the median progression free survival was 17.4 months (range: 2.7–70.0) and local tumor control was achieved in 96.7% of patients. Adverse events were limited to 8.3% of patients, with one grade 4 toxicity within 6 weeks after RRS therapy. RRS is a safe and effective treatment option in selected patients with metastatic RCC in a multimodal approach. Further research is warranted to confirm our findings prospectively.

## 1. Introduction

Renal cell carcinoma (RCC) accounts for 4% of new cancer cases in the US and is responsible for 2% of cancer deaths [1]. For non-metastatic disease, the treatment option of choice is partial or radical nephrectomy. As soon as RCC presents as a metastasized disease, systemic treatment is required [2]. The most frequent sites of metastases in RCC are the lungs and bones. Lymph nodes are the third most frequent site of metastases in 21.8% of all patients with metastatic renal cell carcinoma (mRCC). A total of 20.3% of patients with mRCC reveal metastases of the liver (20.3%) and 8.9% of the adrenal glands [3]. Systemic treatment options for mRCC have changed dramatically during the past few years, and with the approval of immunotherapy based regimes such as Nivolumab in second line settings [4] and, more recently, Nivolumab with Ipilimumab [5] and Pembrolizumab with Axitinib as first line therapies [6], a new standard of care has been gained.

However, the treatment of oligometastatic disease and patients with mixed responses to systemic therapy remains challenging. In selected patients, local therapy of metastases seems to be a therapeutic option [7]. Conversely, radiotherapy for local treatment has not been applied in RCC for a long time as high radioresistance of RCC cell lines has been observed in vitro [8]. Although other groups demonstrated the radiosensibility of RCC [9], conventional radiotherapy is mostly limited to palliation of symptoms, as ablative doses are high and are accompanied by considerable toxicity [10].

Robotic radiosurgery (RRS), as a variant of stereotactic radiotherapy, has gained interest as a method to deliver high, ablative radiation doses in recent years [11]. Initially introduced for the treatment of brain tumors such as metastases, meningeomas or vestibular schwannomas, RRS treatment has recently also been applied for solid tumors throughout the body, as long as the lesion can be clearly visualized and can be approached with an accuracy of about 1 mm [12]. In non-metastatic RCC, RRS treatment has shown local tumor control rates of 98% with high safety [13].

However, the safety and efficacy of RRS for visceral and lymph node metastases of RCC is unclear. Therefore, we aim to investigate local tumor control, progression-free survival (PFS) and overall survival (OS) as well as adverse event in patients with mRCC who have undergone RRS.

## 2. Results

Between December 2005 and September 2019, 60 patients with metastatic RCC undergoing RRS were included in this study. From this cohort, 44 patients presented with visceral metastasis (73.3%) and 16 with lymph node metastasis (26.7%). The median age of all patients at the time of RRS treatment was 64 years (range 42–83), whereas the median age at diagnosis was 56 years (range 37–81), see Table 1. In both groups, the majority of patients were male (75% vs. 70%, *p* = 0.730), had a favorable international metastatic renal cell carcinoma database (IMDC) risk (61.4% vs. 50.0%) and presented with clear cell histology (86.4% vs. 93.8%). The median follow-up time was 22.1 months (range 2.9–108.2).

Patients with visceral metastases received significantly less fractions (median 1, range 1–5) compared to patients with lymph node metastases (median 1, range 1–5 *p* = 0.003). The prescription dose for visceral metastases (median 24 Gy, range 8–26) was significantly higher than the prescription dose for lymph node metastases (median 18 Gy, range 7–26, *p* < 0.001). Prescription isodose (median 70 Gy, range 60–75 versus median 70, range 65–70), target volume (median 26.3 cm^3^, range 1.4–97.4 versus median 18.6 cm^3^, range 2.9–120) and number of treated metastases (median 1, range 1–2 versus median 1, range 1–2) did not differ significantly between both groups (Table 2).

**Figure 1:**
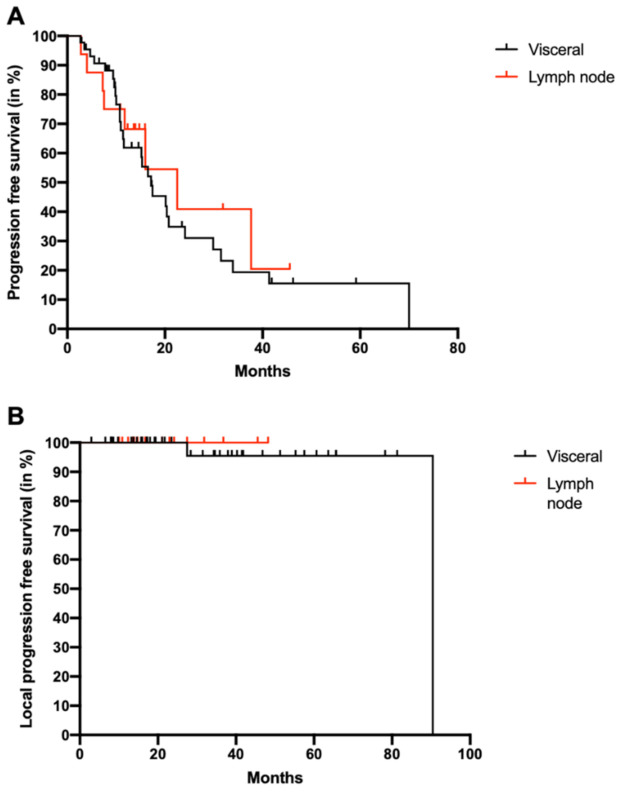
Progression free survival and local tumor control after RRS. (**A**): Progression free survival was calculated for patients with lymph node metastases (red) and visceral metastases (black) treated with RRS therapies. (**B**): Local progression free survival was determined as recurrence within the area of the RRS. Again, patients with lymph node metastases (red) and visceral metastases (black) treated with RRS therapies were analyzed separately. RRS: robotic radiosurgery.

The median progression free survival (PFS) after RRS treatment was 17.4 months (range: 2.7–70.0). The PFS of patients with lymph node metastases (22.5, range 2.8–45.6) was not significantly different from the PFS of patients with visceral metastases (17.2 months, 2.7–70.0, *p* = 0.595) (Figure 1A). Local tumor control was achieved in 96.7% of patients, with only two patients progressing locally in visceral metastases at the radiation site after 2.3 and 7.5 years. There was no progression of lymph node metastases after RRS, but there is no statistically significant difference between both groups (*p* = 0.634) (Figure 1B).

**Figure 2:**
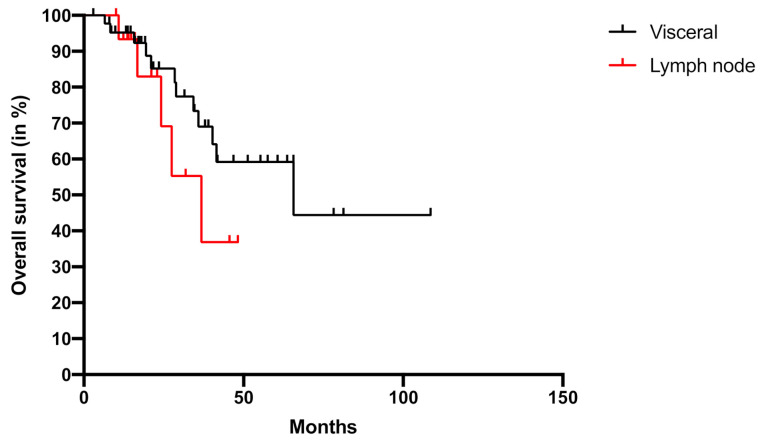
Overall survival after robotic radiosurgery. Overall survival of patients with lymph node (red) and visceral metastases (black) of renal cell carcinoma is calculated with Kaplan–Meier method.

Next, we examined the overall survival (OS) after RRS of all patients included in the study. The median OS was 65.7 (range: 2.9–108.6) months, with 30% of patients being still alive after 3 years. Patients with visceral metastases revealed a median OS of 65.7 months (2.9–108.6) and with lymph node metastases of 36.8 months (10.0–48.2, *p* = 0.258) (Figure 2).

Adverse events during and directly after the procedure have not occurred, as neither bleeding, necrosis nor direct need for intervention were observed. At the 6-week follow up, adverse events were limited to 8.3% of patients, as shown in Table 3. Three patients presented with grade 1 fatigue, one patient with grade 2 fatigue and one patient with grade 4 stroke and thrombosis of the left arm.

## 3. Discussion

Our data are the first to demonstrate the clinically meaningful efficacy of RRS as a local therapy to control visceral and nodal metastases in RCC. The local tumor control rate of 97% resembles the one of surgical procedures [14].

In RCC, radiotherapy with conventional fractions is traditionally applied for palliation and provides sufficient symptom control [15]. Although a high percentage of patients experiences a response in symptoms, the time of duration is limited but dose dependent [16]. In order to increase the delivered doses and overcome the inherent radioresistance of RCC, stereotactic ablative radiotherapy (SABR) has been introduced [10]. However, the application of high doses is still limited by the toxicity of neighboring tissue in the field of the radiation beam and by organ movement. Therefore, image guided robotic radiosurgery with real time tumor tracking and intrafraction movement correction seems to be prone to solve this problem and reaches a high accuracy with highly limited toxicity to neighboring tissue [17].

### 3.1. RRS in Primary RCC

As shown by Correa et al. in a systematic review, RRS is an emerging treatment option in primary RCC. Local tumor control was 97.2% across 25 studies [18]. Grade 3 or 4 toxicities are rare and were especially seen in patients with a large tumor volume above 4 cm [19]. Shortly after RRS, renal function remains stable even in patients with preexisting chronic kidney disease (CKD), but might deteriorate in long-term follow up, as seen in small study cohorts depending on fractions and tumor volume [20]. As RRS is a non-invasive therapy performed in an outpatient setting, it has mainly been used in elderly patients (median 70.4 years, range 62–83) with relevant comorbidities and a high risk of CKD progression [18]. In contrast, the patient cohort in our study is 6 years younger, thereby strengthening the fact that in metastatic RCC there might not be a focus on fragile patients, but on patients still being fit for therapy and being treated by RRS in a multimodal approach.

### 3.2. RRS in Metastatic RCC

To date, RRS as a treatment option for metastatic RCC has mainly focused on cranial and spinal metastases. RRS using the CyberKnife^®^ system has been established as a substitute for brain and spinal surgery in high risk patients [21]. In these clinically relevant locations, local tumor control rates of 98% after 15 months were observed [22]. As most studies focusing on metastatic RCC have used heterogenous cohorts including bone, visceral and lymph node metastases in one cohort [23], direct comparisons are limited. RRS of bone metastases originating from RCC resulted in a 3-year local progression free survival of 88% when high dose regimes were used [24]. In contrast, a heterogenous patient cohort with mainly bone metastases from RCC showed local recurrence in only 2% [25]. Another meta-analysis across 28 studies with stereotactic ablative radiation therapy in oligometastatic RCC patients revealed a median one year local tumor control rate of 89.1% for extracranial metastases [26]. Grade 3 and 4 toxicity was observed in 0–5% of patients across all studies [23]. In line with the literature, we observed a comparable local progression free survival rate and side effects in our study. Side effects were especially limited as only one patient experienced grade 4 toxicity and required hospitalization as well as thrombectomy. This adverse event might be caused by comorbidities such as atrial fibrillation rather than RRS.

Compared to metastasectomy, RRS is less invasive and might be applied in other settings than metastasectomy. As lymph node metastases without other manifestations are rare, few studies have focused on metastasectomy in this patient collective [27]. For visceral organs, metastasectomy of the pancreas seems to be beneficial in fit patients, but it is associated with high in-hospital mortality [28]. In patients with metastases of the liver, metastasectomy is associated with a higher overall survival. However, patients with synchronous metastases do not profit from this procedure [29].

The median age of patients undergoing RRS for oligometastatic disease is 62 across 28 studies in a meta-analysis [26], as RRS is not used to treat fragile patients as for primary RCC. Our patient cohort is 2 years older and the majority are favorable risk patients according to the IMDC risk classification.

### 3.3. RRS as Combination Therapy

Most metastatic RCC patients undergoing RRS are simultaneously treated with either systemic antiangiogenic or immune-modulating agents. Thus, combinatory effects on efficacy and side effects have to be discussed. So far, RRS in patients on antiangiogenic therapy based on tyrosine kinase inhibitors has been demonstrated to be effective with almost no additional toxicity [22]. Immunogenic effects of radiotherapy have been described previously and abscopal effects have been reported, gaining further interest in the era of immunotherapy [30,31]. In melanoma, these abscopal effects have been reported when RRS was administered prior or concomitant to immunotherapy [32]. However, clear evidence from prospective studies is still missing [33]. In our study, we have not observed abscopal effects. However, the study cohort enclosed only 60 patients and not all received additional systemic therapy. Recently, the first data derived from the study of Hammer et al. (NCT03065179) revealed an objective response rate of 55% using the combination of radiotherapy and nivolumab and ipilimumab in standard dosing [34]. Compared with the 42% objective response rate of Nivolumab with Ipilimumab in the CheckMate 214 study [5], this approach seems to be promising, but requires further research as Hammer et al. only included 25 patients in their study.

Contrary to primary RCC where RRS is used in fragile patients at high risk for complications and death after surgery, RRS might be used as an additive therapy in metastatic RCC more frequently in younger and healthier patients in order to prolong progression free survival times of their systemic therapy lines. Various clinical trials are therefore ongoing to elucidate the efficacy of combinations of immunotherapy and RRS (NCT03825510, NCT03961971) and the role of RRS in oligo-progressive disease under systemic therapy (NCT03696277).

### 3.4. Limitations

The study is limited by its retrospective study design. Our data can only report on the safety and efficacy of RRS, but future trials will have to demonstrate a PFS and OS benefit when treating oligo-progressive disease or mixed-responses. Furthermore, mainly patients with clear cell histology were included due to the expected prevalence. Studies focusing on neglected histologic subtypes are therefore warranted.

## 4. Materials and Methods

We retrospectively analyzed patients with metastatic RCC undergoing RRS between December 2005 and September 2019. All RRS treatments were performed in an outpatient setting. Inclusion criteria were patients with histologically confirmed metastatic RCC. All patients required radiologically confirmed visceral or lymph node metastases and RRS treatment of the respective metastases. Visceral metastases included organ metastases of the liver, pancreas, spleen and adrenal gland. RRS was indicated on a patient-by-patient decision. Patients with oligometastatic disease, oligoprogressive disease or mixed responses under systemic treatment were included. RRS treatment was performed for progressing metastases only.

Prior to RRS treatment, the precise size and location of metastases were determined by computed tomography (CT) or magnetic resonance imaging (MRI) and patients were evaluated for treatment. Evaluation included prior radiation therapy and the localization of metastases and neighboring tissue at risk of radiotoxicity. Radiotoxicity for neighboring tissues such as stomach, esophagus and bowels was calculated. Metastases up to 3 cm mainly received a single fraction radiation—between 3 and 5 cm, two fractions, and larger than 5 cm, 5 fractions. Therapy decisions were performed on a single patient level.

After selection for eligibility for RRS treatment, cost coverage was checked with the health insurance provider and either granted as part of general agreements or on single request.

Systemic therapy including tyrosine kinase inhibitors and immunotherapy was continued after RRS in patients who had already received systemic therapy prior to treatment and revealed oligoprogressive disease or mixed responses.

Patient follow-up was performed directly after RRS treatment and 6 weeks after treatment for three months after RRS treatment according to the European Association of Urology (EAU) guidelines [35]. Adverse events were classified by the common terminology criteria for adverse events (CTCAE) [36]. RCC risk classification was performed according to the International Metastatic RCC Database Consortium (IMDC) risk criteria [37].

RRS was performed using the Cyberknife robotic radiosurgery system (Accuray Inc., Sunnyvale, CA, USA). Thereby, a 6-MV linear accelerator is brought into treatment position by a high precision six-axis robotic arm and provides between 100 and 120 radiation beams from 270 degrees around the body in a single session. Organ movement is detected by x-ray cameras to adjust for breathing and movement [17]. RRS treatment has not changed significantly over the study time frame. However, due to technological modifications, the duration of one session has been reduced by 50% from approximately 60 min in 2005 to 30 min in 2019.

The Kaplan–Meier method was used to calculate overall survival (OS) and progression free survival (PFS). The Mann–Whitney U Test and Chi-Square test were performed to test for differences between groups. All calculations were performed by Graphpad Prism Software (Version 8.0, San Diego, CA, USA).

Ethical approval was received for this study by the local ethics authorities (Ethikkomission der Ludwig-Maximilian-Universität München, reference number: 20-1092).

## 5. Conclusions

RRS treatment is a highly effective and safe treatment option for patients with metastatic RCC with visceral or lymph node metastases in highly selected cases. Local tumor control is excellent. Prospective trials are warranted to elucidate the role of RRS in combination with checkpoint or tyrosine kinase inhibitory therapy, especially in oligo-progressive disease and mixed responses.

## Figures and Tables

**Table 1:** Patient characteristics.

Parameter	Visceral Metastases		Lymph Node Metastases		*p* Value
	(*n* = 44)		(*n* = 16)		
Age at diagnosis					0.275
Median (Years)	55		60		
Range (Years)	37–77		38–81		
Age at RRS treatment					0.996
Median (Years)	64		64		
Range (Years)	45–80		42–83		
	%	*n*	%	*n*	
Gender					0.730
Male	70	31	75	12	
Female	30	13	25	4	
IMDC					0.731
Favorable	61	27	50	8	
Intermediate	34	15	44	7	
Poor	5	2	6	1	
Histology					0.171
Clear cell	86	38	94	15	
Papillary Typ 2	7	3	0	0	
Chromophob	7	3	0	0	
TFE-3 Translocation	0	0	6	1	
Prior therapies					0.939
Surgery	100	44	100	16	
TKI	36	16	31	5	
Immunotherapy	16	7	13	2	
Therapy at RRS					0.934
No systemic therapy	59	26	63	10	
TKI	25	11	25	4	
Immunotherapy	16	7	13	2	

Percentages may not total 100% due to rounding. RRS: robotic radiosurgery, IMDC: international metastatic renal cell carcinoma database, TFE-3: transcription factor E3, TKI: tyrosine kinase inhibitor.

**Table 2:** Robotic radiosurgery treatment parameters.

Parameter	Visceral Metastases		Lymph Node Metastases		*p* Value
	(*n* = 44)		(*n* = 16)		
	Median	Range	Median	Range	
Fractions	1	1–5	1	1–5	0.003
Prescription Dose (Gy)	24	8–26	18	7–26	<0.001
Prescription Isodose (Gy)	70	60–75	70	65–70	0.434
Target volume cm^3^	26.3	1.4–97.4	18.6	2.9–120	0.434
Number of metastases	1	1–2	1	1–2	0.170

**Table 3:** Adverse events after robotic radiosurgery (within 6 weeks).

Adverse Event	CTCAE 1	CTCAE 2	CTCAE 3	CTCAE >3
Fatigue	3	1	0	0
Stroke/Thrombosis	0	0	0	1

CTCAE: Common Terminology Criteria for Adverse Events.

## Data Availability

The data presented in this study are available on request from the corresponding author.
